# Editorial: Exploring physical activity as a complementary strategy in managing mental illness

**DOI:** 10.3389/fspor.2026.1966365

**Published:** 2026-09-04

**Authors:** Shanshan Li, Zichao Chen, Xinglong Yang, Jason H. Huang

**Affiliations:** 1 Sichuan University, Chengdu, China; 2 The First Affiliated Hospital of Kunming Medical University, Kunming, China; 3 Department of Neurosurgery, Baylor Scott & White Health, Temple, TX, United States

**Keywords:** exercise intervention, mechanisms of exercise therapy, mental health, neuroplasticity, psychiatric treatment

Mental illness remains a major contributor to disability worldwide despite substantial advances in pharmacotherapy and psychotherapy. This persistent burden has intensified interest in complementary strategies that are accessible, scalable, and capable of improving both mental and physical health. Physical activity is particularly compelling in this regard. Beyond its established cardiovascular and metabolic benefits, exercise may influence mental health through multiple interacting pathways, including neuroplasticity, neurotransmitter regulation, neuroendocrine responses, inflammation, sleep, cognition, self-efficacy, and social engagement. Importantly, physical activity should not be viewed as a single intervention: modality, intensity, duration, setting, and individual characteristics may all shape its therapeutic effects.

This Research Topic, “*Exploring physical activity as a complementary strategy in managing mental illness*,” brings together five articles that illustrate this multidimensional relationship. Collectively, they extend the discussion beyond the general question of whether exercise is beneficial and toward more clinically relevant questions: *Which forms of physical activity are effective? For whom? At what intensity? Through what interacting behavioral and physiological pathways? And how might exercise be incorporated into prevention and treatment?*

Two contributions focus particularly on students and young adults, a population in whom psychological distress is common and prevention may have substantial long-term impact. Sun et al., in “*Tai Chi as a preventive intervention for improving mental and physical health in non-depressed college students with high perceived stress*,” evaluated a 16-week supervised Tai Chi intervention in students experiencing high perceived stress but without established depression. Tai Chi produced improvements across several domains, including perceived stress, sleep quality, somatic anxiety, fatigue, social functioning, and aspects of physical fitness. The study is noteworthy for shifting attention upstream—from treatment of established psychiatric disease toward intervention in an at-risk population. It also demonstrates the potential value of mind–body approaches that integrate movement, attentional control, breathing, and behavioral regulation.

Complementing these findings, Sveaas et al., in “*Effects of exercise on depression and anxiety in university students: a systematic review and meta-analysis*,” synthesized evidence regarding cardiorespiratory and muscular-strength exercise among higher-education students experiencing mental health challenges. Their findings suggest medium-to-large improvements in depressive and anxiety symptoms, supporting exercise as a potentially useful component of student mental health services. Together, these two studies suggest a continuum of opportunity: physical activity may have value both before the development of a defined psychiatric disorder and as part of its management once symptoms emerge.

The question of exercise “dose” is addressed more directly by Zeng and Wang in “*The impact of high-intensity exercise on patients with depression: a systematic review and meta-analysis of randomized controlled trials*.” Their analysis suggests that high-intensity exercise can modestly improve depressive symptoms, while also highlighting heterogeneity across outcome measures, exercise modalities, intervention durations, and patient populations. Longer interventions appeared more beneficial, and effects differed according to age and exercise type. These findings underscore an important principle for the field: exercise prescriptions for mental health should ultimately become more individualized, rather than relying on a uniform recommendation that more activity is necessarily better.

The importance of modality is further illustrated by Chen et al. in “*The effects of taekwondo on depression symptoms and cognitive function: a systematic review and meta-analysis*.” Their analysis found meaningful improvements in both depressive symptoms and cognitive function (Chen et al.). Taekwondo is especially interesting because its potential effects extend beyond physical exertion alone; it incorporates coordination, sustained attention, learning, discipline, and social interaction. Such multidimensional interventions may engage several mechanisms simultaneously and reinforce the concept that the psychological context in which physical activity occurs can be as important as its physiological intensity.

Finally, Clemente-Suárez et al., in “*Bidirectional effects of physical activity and sleep on health: evidence and future directions*,” broaden the conceptual framework by emphasizing the reciprocal relationship between physical activity and sleep. Exercise can improve sleep quality, whereas inadequate or disrupted sleep can impair recovery, performance, motivation, and continued participation in physical activity. Both behaviors influence mental health, and their effects are shaped by exercise timing and intensity, chronotype, nutrition, and individual characteristics. This bidirectionality illustrates why physical activity should be considered within a broader behavioral and biological system rather than as an isolated therapeutic exposure.

Taken together, the articles in this Research Topic support physical activity as a promising complementary strategy across the spectrum from mental health promotion and prevention to management of established depression and anxiety. At the same time, they caution against oversimplification. “Exercise” encompasses diverse interventions, and therapeutic responses are likely moderated by dose, modality, age, baseline mental health, sleep, adherence, social context, and individual preference.

The next phase of research should therefore move from demonstrating general efficacy toward precision exercise interventions for mental health. Larger and methodologically rigorous trials are needed to define dose-response relationships, compare modalities, identify predictors of response, establish durability of benefit, and determine how physical activity can best complement pharmacological and psychotherapeutic treatments. Mechanistic studies integrating neurobiological, inflammatory, cognitive, sleep, and behavioral measures may help explain why particular interventions benefit particular individuals. Equally important will be pragmatic studies addressing adherence, accessibility, implementation, and the potential consequences of excessive exercise.

Physical activity is unlikely to replace established psychiatric treatments, nor should it be presented as a universal prescription. Rather, the evidence assembled in this Research Topic supports a more integrated view: appropriately selected physical activity can become one component of personalized, multidisciplinary mental healthcare—one that simultaneously engages the brain, body, behavior, and social environment. Advancing this framework may help transform exercise from a broadly recommended healthy behavior into a more precisely understood and effectively deployed complementary strategy for promoting mental health.

